# The use of chitosan oligosaccharide to improve artemisinin yield in well-watered and drought-stressed plants

**DOI:** 10.3389/fpls.2023.1200898

**Published:** 2023-06-02

**Authors:** Ana L. García-García, Ana Rita Matos, Eduardo Feijão, Ricardo Cruz de Carvalho, Alicia Boto, Jorge Marques da Silva, David Jiménez-Arias

**Affiliations:** ^1^ Grupo Síntesis de Fármacos y Compuestos Bioactivos, Departamento de Química de Productos Naturales y Sintéticos Bioactivos, Instituto de Productos Naturales y Agrobiología, Consejo Superior de Investigaciones Científicas, San Cristóbal de La Laguna, Spain; ^2^ Programa de Doctorado de Química e Ingeniería Química, Universidad de La Laguna, San Cristóbal de La Laguna, Spain; ^3^ Departamento de Biologia Vegetal, Faculdade de Ciências da Universidade de Lisboa, Lisbon, Portugal; ^4^ BioISI - Biosystems and Integrative Sciences Institute, Plant Functional Genomics Group, Departamento de Biologia Vegetal, Faculdade de Ciências da Universidade de Lisboa, Lisbon, Portugal; ^5^ MARE - Marine and Environmental Sciences Centre and ARNET – Aquatic Research Infrastructure Network Associate Laboratory, Faculdade de Ciências da Universidade de Lisboa, Lisbon, Portugal; ^6^ Centre for Ecology, Evolution and Environmental Changes (cE3c), Faculdade de Ciências, Universidade de Lisboa, Lisbon, Portugal; ^7^ ISOPlexis—Center for Sustainable Agriculture and Food Technology, Madeira University, Funchal, Portugal

**Keywords:** chitosan oligosaccharide, artemisinin, *Artemisia annua*, drought, elicitation, stress, bioactive metabolites

## Abstract

**Introduction:**

Artemisinin is a secondary metabolite well-known for its use in the treatment of malaria. It also displays other antimicrobial activities which further increase its interest. At present, Artemisia annua is the sole commercial source of the substance, and its production is limited, leading to a global deficit in supply. Furthermore, the cultivation of A. annua is being threatened by climate change. Specifically, drought stress is a major concern for plant development and productivity, but, on the other hand, moderate stress levels can elicit the production of secondary metabolites, with a putative synergistic interaction with elicitors such as chitosan oligosaccharides (COS). Therefore, the development of strategies to increase yield has prompted much interest. With this aim, the effects on artemisinin production under drought stress and treatment with COS, as well as physiological changes in A. annua plants are presented in this study.

**Methods:**

Plants were separated into two groups, well-watered (WW) and drought-stressed (DS) plants, and in each group, four concentrations of COS were applied (0, 50,100 and 200 mg•L-1). Afterwards, water stress was imposed by withholding irrigation for 9 days.

**Results:**

Therefore, when A. annua was well watered, COS did not improve plant growth, and the upregulation of antioxidant enzymes hindered the production of artemisinin. On the other hand, during drought stress, COS treatment did not alleviate the decline in growth at any concentration tested. However, higher doses improved the water status since leaf water potential (YL) improved by 50.64% and relative water content (RWC) by 33.84% compared to DS plants without COS treatment. Moreover, the combination of COS and drought stress caused damage to the plant’s antioxidant enzyme defence, particularly APX and GR, and reduced the amount of phenols and flavonoids. This resulted in increased ROS production and enhanced artemisinin content by 34.40% in DS plants treated with 200 mg•L-1 COS, compared to control plants.

**Conclusion:**

These findings underscore the critical role of ROS in artemisinin biosynthesis and suggest that COS treatment may boost artemisinin yield in crop production, even under drought conditions.

## 1 Introduction

Sweet wormwood (*Artemisia annua* L.) is an annual herb that produce artemisinin in its trichomes. Artemisinin is an endoperoxide sesquiterpene lactone which is used in malaria treatment. Globally, between 2000 and 2020, approximately 1.7 billion cases of malaria and 10.6 million malaria-related deaths were reported (WHO, 2021). Artemisinin-based combination therapies (ACTs) remain efficacious against *Plasmodium* species (malaria’s parasite). Despite the creation of a malaria vaccine, which the World Health Organization (WHO) recommended for use in preventing *P. falciparum* malaria among children residing in areas with moderate to high transmission rates, ACTs are the first- or second-line malaria treatment (WHO, 2021). Furthermore, artemisinin has activity against several type of cancers (Efferth, 2006) and Li et al. (2005) found that the plant extract has anti-viral activity against Severe Acute Respiratory Syndrome-associated coronavirus (SARS-CoV).

Besides, *A. annua* crops have the potential to provide low-cost raw materials to pharmaceutical industries and commercially support farmers engaged in large-scale production (Soni et al., 2022). Since, the only commercial source of artemisinin is the *A. annua* plant, the chemical synthesis has low yields and the process is long and relatively complex (Wang et al., 2014), artemisinin production is a key challenge in the artemisinin supply chain (Lee et al., 2023). Thus, raising the artemisinin content of *A. annua* is a viable strategy for lowering the cost of artemisinin while increasing the availability of ACTs.

The most significant and harmful form of stress to plant production worldwide is drought, which diminishes yields at various crucial stages throughout the crop cycle (Jacques et al., 2021; Agathokleous et al., 2022). In *A. annua*, artemisinin content decreased due to water stress (Marchese et al., 2010; Yadav et al., 2014; Soni and Abdin, 2017). Specifically, Yadav et al. (2014) found that mild and moderated water stress reduced artemisinin content, plant growth, development, and physiological status, as well as glandular trichomes density and size, in all the plant development stages (from the early vegetative stage to full bloom stage). However, in other reports different results were described. Soni and Abdin (2017) found slightly higher artemisinin amounts in severely stressed plants at the preflowering and flowering stages. Additionally, Marchese et al. (2010) quantified artemisinin content after 14, 38, 62 and 86 h of water deficit and obtained a significative increase (29%) with moderate water deficit (38 h). These studies highlight the need to further investigate how drought affects artemisinin production.

Drought stress also has detrimental effects on physiological, morphological and biochemical processes. These aspects were deeply discussed by Farooq et al. (2012) and Yang et al. (2021). The authors point out how growth parameters are negatively affected due to a decrease in cell expansion and energy supply, loss of turgor and impaired enzyme activity. To alleviate these effects, plants activate their stress response mechanisms, which includes the expression of functional gene products (such as aquaporins, proline, soluble sugars, etc.), regulatory gene products (such as mitogen-activated protein kinases) which can cause changes in plant morphology or physiology, and the synthesis of hormones (such as abscisic acid) (Yang et al., 2021). Most of these mechanisms serve to regulate plant water status through osmotic regulation and plant transpiration. These, as well as the antioxidant machinery and drought-induced proteins that help the plant to face drought stress are reviewed in depth by Yang et al. (2021).

Plant water relations are characterised by key features such as leaf water potential, osmotic potential, pressure potential, and relative water content. (Kirkham, 2005). In fact, the study of water stress commonly employs leaf water potential (Ψ_L_) and relative water content (RWC) as these are significantly impacted by water deficiency (Farooq et al., 2012). Besides, drought can increase the production of reactive oxygen species (ROS), such as hydrogen peroxide (H_2_O_2_), and generate oxidative damages (Ahluwalia et al., 2021). These damages could affect different plant processes since it induces protein denaturation and loss of enzyme activity, lipid peroxidation (which affects membrane structure), and DNA strand breakage (Yang et al., 2021).

Plants have two types of antioxidant protection systems, enzymatic and non-enzymatic, to maintain a moderate level of ROS which at low levels can act as secondary messengers (Kirova et al., 2021). The non-enzymatic antioxidant system consists of substances, such as phenols and flavonoids (Kirova et al., 2021). On the other hand, catalase (CAT), superoxide dismutase (SOD), ascorbate peroxidase (APX) and glutathione reductase (GR) are some of the main antioxidant enzymes (Yang et al., 2021). However, a largely unexplored aspect of the response to plant stress is the fatty acid profile changes. These are the main components of cell membranes as well as precursors of several signalling molecules, involved in moisture and heat loss prevention, and are associated with cell identification specificity and tissue immunity (Gu et al., 2020).

A well-known strategy to face different stresses and/or improve crop production is the use of biostimulants, since they promote plant growth and trigger natural defences (García-García et al., 2020). Chitosan and their derivatives have been widely studied due to their properties as biostimulants (García-García et al., 2020). It is also an abundant polymer that can be obtained from all crustaceans and insects shells, and in certain fungi, algae and yeast (Rinaudo, 2006). Chitosan oligosaccharides (COS) are chitosan degradation products obtained through physical, enzymatic, or chemical hydrolysis (Naveed et al., 2019; Yuan et al., 2019). Furthermore, COS have a lower molecular weight, a lower degree of polymerization, a higher degree of deacetylation, less viscosity, more solubility, biodegradability, biocompatibility, and exposed N-glucosamine units than chitosan and chitin (Benchamas et al., 2021). Besides, COS have been shown to have a variety of effects on plant cultivation, including inducing resistance to plant diseases, stimulating plant growth and development, and enhancing the yield and quality of plant products (Yuan et al., 2019).

Moreover, several studies showed that the use of COS is a promising option to increase the production of secondary metabolites with pharmacological significance. Thus, COS improved the pharmaceutical value of *Catharanthus roseus* (L.) G. Don by increasing the content of vindoline and catharanthine in the leaves, and by increasing the fresh weight of roots, stems and leaves (Tang et al., 2022). Moreover, greek oregano (*Origanum vulgare* ssp. hirtum (Link) Ietsw.) treated with 200 and 500 ppm of COS displayed superior plant height, while the use of 50 and 200 ppm COS doses significantly increased polyphenol content (Yin et al., 2012a). Moreover, COS increased the fresh and dry weight of mint, as well as secondary metabolites production (Ahmad et al., 2017). Jaleel et al. (2017) also found an enhancement in biomass parameters and an increased yield of essential oil and citral content in lemongrass (*Cymbopogon flexuosus* (Steud.) Wats) treated with COS. Foliar spray with COS improved shoot fresh weight, shoot dry weight and fruit fresh weight of chili (*Capsicum frutescens* L.) plants (Dzung et al., 2017). Promising results were also obtained with COS-treated wheat seedlings, where growth parameters improved (Zhang et al., 2016). Also, COS treatment afforded better yield and increased superoxide dismutase activity (SOD) and peroxidase (POD) activity in tea (*Camellia sinensis* (L.) O. Kuntze) plants (Ou et al., 2022). Besides, chitosan oligomers treatment increased growth, mineral uptake and yield in coffee (*Coffea canephora* Pierre ex A.Froehner) plants (Dzung et al., 2011). Furthermore, the authors proposed COS treatment to improve coffee drought resistance since it reduced the transpiration of leaves.

In fact, it has been reported a reduction in plant transpiration with chitosan and COS treatment in several species (Lee et al., 1999; Bittelli et al., 2001; Iriti et al., 2009; Khokon et al., 2010; Dzung et al., 2011; Pongprayoon et al., 2013; Mirajkar et al., 2019). This is a common strategy to avoid water loss under drought stress, and therefore, chitosan and its derivatives have been proposed to increase drought tolerance, displaying excellent results in different species. Thus, COS addition to Buddhist Pine (*Podocarpus macrophyllus* (Thunb) Sweet) enhanced drought tolerance by causing starch allocation towards roots, where δ^13^C abundance (used to quantify newly formed photosynthates in trees) and antioxidant enzyme activity were upregulated (He et al., 2020). Muley et al. (2019) reported that 75 and 50 mg · L^-1^ of chitosan and COS, respectively, were effective to improve drought tolerance in potato (*Solanum tuberosum*, L.). In cowpea (*Vigna unguiculata* (L) Walp), treatment with 250 mg · L^-1^ of chitosan increased plant growth and yield under normal and drought stress conditions (Farouk and Amany, 2012). Besides, the treatment of apple (*Malus sieversii* (Ledeb.)M.Roem) trees with chitosan and COS has increased their drought tolerance (Yang et al., 2009), and similar effects have been observed in maize (*Zea mays* L.) (Rabêlo et al., 2019), sugarcane (*Saccharum* hybrid cultivar) (Mirajkar et al., 2019), barley (*Hordeum vulgare* L.) (Hafez et al., 2020) and in two basil species (*Ocimum ciliatum* Hornem and *O. basilicum* L.) (Pirbalouti et al., 2017). This superior resistance has been associated with an improvement in osmotic adjustment and antioxidant systems.

Furthermore, COS seems to be a promising option to enhance the production of secondary metabolites and biomass as well as drought tolerance. Specifically, after 48 h of treating *A. annua* with 100 mg · L^-1^ chitosan, a significant increase in artemisinin production was observed (Lei et al., 2011). Also, in two species of basil, the highest total phenolic content was obtained by foliar spraying chitosan (0.4 g·L^-1^) under low watering or mild drought stress (Pirbalouti et al., 2017). In summer savory (*Satureja hortensis* L.) the interaction between chitosan treatment and drought stress also increased total phenolic content (Alizadeh et al., 2020). Thus, our current study analyses the effect of different COS concentrations, drought stress and their interactions in growth, drought stress tolerance and artemisinin production of *A. annua* plants with the aim of artemisinin production optimisation.

## 2 Materials and methods

### 2.1 Plant material

*Artemisia annua* seeds (purchased from East-West Seed International) were sown in the seedbed and kept at 16-h light photoperiod at 400 μmol·m^−1^·s^−1^, 60% of relative humidity, 25°C/20 °C (day/night). One month after sowing, seedlings of similar size were transplanted to 1 L pots (one per pot) and placed in a growth chamber under the mentioned conditions. In all cases, a commercial soil was used (ECOGrow® universal soil). Every other day, the plantlets were watered using tap water. The experiment was carried out twice during the vegetative stage, specifically, stage E established by Marchese et al. (2023).

### 2.2 Chitosan oligosaccharide treatment and water stress imposition

Twenty days after transplanting (vegetative stage E), chitosan oligosaccharides (purchased to TCI Europe N.V.) were applied, by spraying, randomly chosen plants. Different concentrations with 0.02% tween20 were tested (50,100 and 200 mg·L^-1^) and distilled water was used as a control. Each plant was sprayed with 100 ml of each treatment. Afterwards, plants were watered to field capacity and divided in two groups: well-watered (WW) and drought-stressed (DS) plants. Thus, the groups were: Well-watered control plants (WW-Control), drought-stress control plants (DS-Control), well-watered plants treated with 50 mg·L^-1^ (WW-COS50), 100 mg·L^-1^ (WW-COS100) and 200 mg·L^-1^ (WW-COS200), as well as, drought-stressed plants treated with 50 mg·L^-1^ (DS-COS50), 100 mg·L^-1^ (DS-COS100) and 200 mg·L^-1^ (DS-COS200).

WW-plants were maintained at ~ 100% of soil water content (SWC). Progressive drought stress was imposed by withholding irrigation, reaching 47.98 ± 0.98% of SWC after 9 days ( $\bar{\text{X}}$ ± SE, **Supplementary S.1**). Soil water content was calculated according to Barradas et al. (2021), where SFW is the soil fresh weight (pot weight at the sampling time) and SFC is the soil weight at field capacity (pot weight before stress imposition):


$$SWC=\;\frac{SFW}{SFC}\;\times 100$$

The duration of water stress was determined by monitoring the ratio Fv/Fm, which quantifies the maximum photosystem II quantum efficiency. This value ranges from 0.75 to 0.85 in non-stressed plants and a declining of these values are observed when plants are exposed to stress, such as drought (Björkman and Demmig, 1987; Bolhar-Nordenkampf et al., 1989; Maxwell and Johnson, 2000). Accordingly, after 9 days, a reduction of 2.97% was observed in drought-stressed plants (**Supplementary S.2**). The values Fv/Fm were measured with Handy PEA fluorimeter (Hansatech, Kings Lynn, UK).

### 2.3 Water potential, relative water content and biomass

At the end of the experiment, mature leaves were excised for leaf water potential (Ψ_L_) measurement. A Scholander pressure chamber (PMS Instrument Company, model 600D, USA) was used. Each measurement was the mean value of six samples. Besides, fully developed leaves were cut and weighed promptly to obtain their fresh weight (FW). The leaves were then immersed in deionized water for 24 hours before being re-weighed to obtain their fully turgid weight (TW). Subsequently, the leaves were dried at 38°C and weighed to calculate their dry weight (DW). The leaf RWC (relative water content) was calculated as:


$$RWC=\;\frac{FW-DW}{TW-DW}\;\times 100$$

The aerial part was dried at 38 °C for three days and dry weight was determined by adding the previously measured DW of RWC for more precise results.

### 2.4 RGB imaging protocol

Throughout the experiment, seven plants per group were photographed and the number of plant-specific pixels was calculated utilising red-green-blue (RGB) imaging employing high-resolution top-view and side-view RGB cameras and an optimised picture segmentation method for automated analysis.

Plant area (cm^2^) obtained from processed images of the side (area Side) and the top view (area Top), was used to calculate digital biomass (DB) according to Rahaman et al. (2017) and Sorrentino et al. (2022):


$$DB=\;\sqrt{area\;Side^{2}\;\times \;area\;Top}$$

Results of digital biomass at the end of the experiment (DB_d9) were used to run a simple linear regression model to explain the average DB_d9 as a function of the dry weigh average. The model was implemented in R Studio (version 2022.07.1 + 554, © 2009-2022 RStudio, PBC) using the packages ggplot2 and pwr. Then, the relative growth rate (RGR) was calculated according to Sorrentino et al. (2022) where t_1_ and t_2_ represent the period (days), and DB_1_ and DB_2_ represent the equivalent digital biomass:


$$RGR=\;\frac{\ln\left(DB_{2}\right)-\ln\left(DB_{1}\right)}{t_{2}-\;t_{1}}$$

### 2.5 Fatty acid profile

Leaf fatty acid composition (n = 5) was determined by acidic trans-esterification of leaf tissues grounded in liquid nitrogen as previously described (Gameiro et al., 2016; Sebastiana et al., 2018). Fatty acid methyl esters (FAME) were separated in a gas chromatograph (430 Gas Chromatograph, Varian) equipped with a hydrogen flame-ionization detector using a fused silica 0.25 mm i.d. × 50 m capillary column (WCOT Fused Silica, CP-Sil 88 for FAME, Varian). Heptadecanoate (17:0) was used as an internal standard.

### 2.6 Hydrogen peroxide content and antioxidant enzymes activity

Hydrogen peroxide (H_2_O_2_) content was determined with the method described by Zhang et al. (2020) with slight modifications. Each measurement was the mean value of six samples. About 250 mg of fresh leaves were homogenized with 2.5 ml of 0.1% (w/v) trichloroacetic acid (TCA) in an ice bath. The homogenate was centrifuged at 12000 g at 4°C for 30 min, then 0.5 ml of supernatant was taken and mixed with aqueous 10 mM K_3_PO_4_ (pH 7.0, 0.5 ml) and 1 M potassium iodide (KI, 1 ml). The absorbance of the supernatant was measured at 390 nm. A standard curve was used to calculate the H_2_O_2_ content.

To assess enzymatic activity, five samples per group were taken and a quantity of 200 mg of fresh weight was ground with liquid nitrogen using a mortar and pestle to obtain a homogenized sample. the soluble protein fraction was extracted according to Feijão et al. (2022) and centrifuging the samples at 12,000 × g for 15 minutes at 4°C to recover the supernatant. The measurements were carried out at 25°C using an Epoch 2 Microplate Reader (BioTek Instruments, VA, United States). The enzymatic activity of catalase (CAT) was assessed according to Aebi (1984) by monitoring H_2_O_2_ consumption and the resulting decrease in absorbance at 240 nm (ε = 39.4 M^-1^ · cm^-1^). Measurement of ascorbate peroxidase (APX) was performed according to Tiryakioglu et al. (2006) via ascorbate oxidation detection as a reduction in absorbance at 290 nm (ε = 2.8 mM^-1^ · cm^-1^). Superoxide dismutase (SOD) activity was assayed following the method described by Marklund and Marklund (1974) through the measurement of pyrogallol reduction as an increase in absorbance at 325 nm. To facilitate data comparison, pyrogallol autoxidation was measured without any enzymatic extract during the reading period. Glutathione reductase (GR) activity was determined using the glutathione-dependent oxidation of NADPH, and the reduction in its absorbance at 340 nm (ε = 6.22 mM^-1^ · cm^-1^), as described by Edwards et al. (1990). Total protein concentration was determined with a standard curve of bovine serum albumin (BSA) following the method described by Bradford (1976).

### 2.7 Total phenolics and flavonoids quantification

Fresh samples were taken for phenols and flavonoids quantification (n = 6) and were measured according to Meng et al. (2019) with slight modifications. Fresh leaf material (100 mg) was frozen in liquid nitrogen, homogenized in 1 ml of 80% methanol, and sonicated for 30 minutes. Then, the suspension was centrifugated at 10000 × g for 15 minutes and the supernatant was used for quantification.

To quantify total phenolics an aliquot of 100 µl reacted for 2 h in the dark with Folin-Ciocalteu (1:10) reagent and 7.5% of Na_2_CO_3_. The total phenolic concentration was expressed as mg gallic acid equivalent (GAE) · g^-1^ FW after the reaction product was detected measuring absorbance at 765 nm. To determine flavonoids, an aliquot of 200 µl was reacted for 5 minutes with 60 µl of 5% NaNO_2_ in 800 µl of distilled water. Then, 120 µl of 10% Al(NO_3_)_3_ was added to react for 6 min. Finally, 400 µl of 1 M NaOH and 400 µl of distilled water were added to complete the reaction. The total flavonoid content was expressed as mg catechin equivalent (CE) · g^-1^ FW by measuring absorbance at 510 nm. In both cases a calibration curve was made.

### 2.8 Artemisinin quantification

Artemisinin content was quantified according to Nganthoi and Sanatombi (2019) with slight modifications. Measurements were estimated from 6 samples, each one consisting of a pool of 2 plants. Powdered dry leaf samples (750 mg) were macerated at room temperature for 24 h with 10 ml of toluene. The filtrate was vacuum-dried using a rotary evaporator (Rotavapor® R-200, heating bath B-490, Büchi, Switzerland), and the residue was dissolved in 10 ml of extracting solvent. Then, the extract was washed with an equal volume of NaOH (2% w/v) followed by 4 washes with distilled water. To remove coloured pigments, activated charcoal was added to the extract until the colour disappeared. The filtrate was dried under vacuum and the resulting extract was dissolved in 5 mL of methanol. To prepare the standard curve, artemisinin (purity 98%, purchased from Sigma), was dissolved in methanol.

The derivatisation of artemisinin was made with 100 µl of standard artemisinin or plant extract incubated at 50°C for 30 minutes with 800 µl of 0.2% NaOH (w/v). After allowing the solution to cool to room temperature, 0.2 N HCl was added to bring the final volume to 2 ml for measurement by UV- Spectrophotometry.

### 2.9 Statistical analysis

To evaluate the effect of two categorical explanatory variables (irrigation conditions and COS treatment) on the quantitative variables, parametric data were analysed applying two-way ANOVA (p ≤ 0.05), followed by multiple comparisons with Tukey HSD *post hoc* test. A widely accepted notion among biologists is that comparing treatment means is not possible or not advisable when the interaction of two factors is statistically non-significant. However, Wei et al. (2012) showed that when there is no interaction, and there is a significant main effect in, at least, one factor, comparing treatment means could be useful to identify which specific means differ. Owing to this, Tukey HSD *post hoc* test had been performed in several studies when there was not interaction (Jobgen et al., 2009; Lassala et al., 2010; Tan et al., 2010). In this study, Tukey HSD *post hoc* test were made in RGR and artemisinin results for this purpose. For non-parametric data, Kruskal-Wallis’ test (p ≤ 0.05) was applied. The statistical analysis was implemented in R Studio (version 2022.07.1 + 554, © 2009-2022 RStudio, PBC).

## 3 Results

### 3.1 The application of COS treatment increased leaf water potential and reduced relative water content

**Figure 1:**
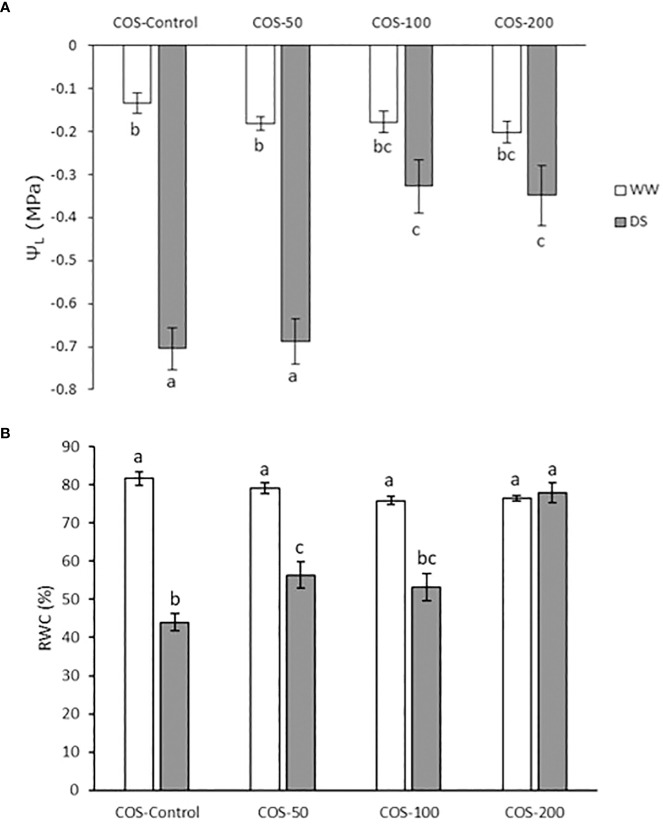
Leaf water potential **(A)** and relative water content **(B)** in well-watered plants (WW) and drought-stressed plants (DS) treated with 0, 50, 100 and 200 mg·L^-1^ of COS. Different letters indicate significant differences according to the Tukey HSD test after two-way ANOVA, p< 0.05. Represented values are the mean ± SE.

Leaf water potential (Ψ_L_) is represented in **Figure 1A**. In non-treated plants, drought stress significantly decreased Ψ_L_. Besides, treatment slightly decreased it in non-stressed plants, but non-significative differences were found between treatments. However, under drought stress, 100 and 200 mg·L^-1^ COS doses increased Ψ_L_. Relative water content (RWC) is represented in **Figure 1B**, where a significant reduction is observed in non-treated plants under drought stress compared with well-irrigated plants. In non-stressed plants, this percentage slightly decreased with non-significative differences between treatments. Under drought stress, COS treatment prevented the reduction of RWC, especially with a 200 mg·L^-1^ dose.

### 3.2 The application of COS treatment did not significantly improve growth parameters

**Figure 2:**
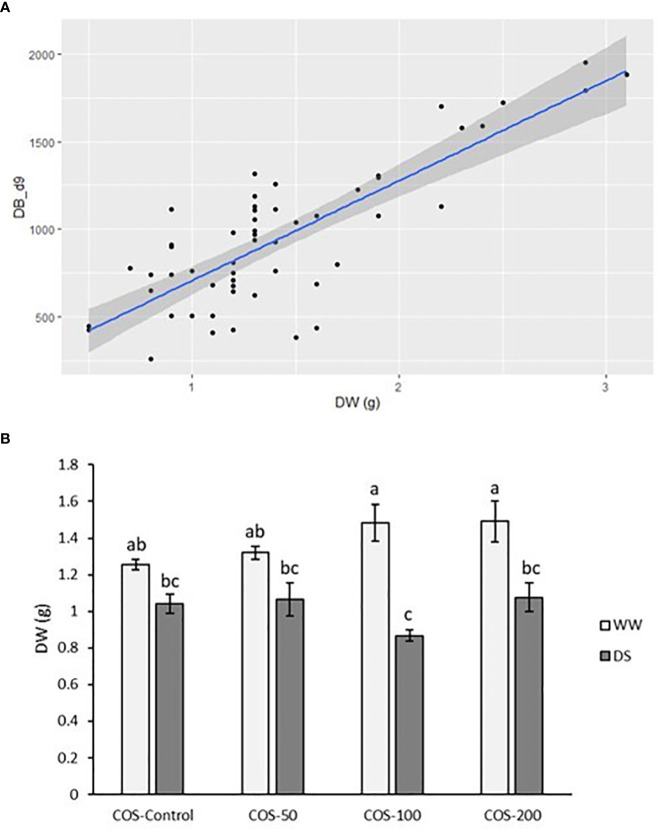
**(A)** Scatter plot between digital biomass at the end of the experiment (DB_d9) and dry weight (DW) at the end of the experiment. **(B)** Dry weight at the end of the experiment in well-watered plants (WW) and drought-stressed plants (DS) treated with 0, 50, 100 and 200 mg·L^-1^ of COS. Different letters indicate significant differences according to the Kruskal-Wallis test, p< 0.05. Represented values are the mean ± SE.

Dry weight and digital biomass at the end of the experiment presented a positive correlation and the fitted model presented a residual standard error of 0.343 (**Figure 2A**). The coefficient of determination is R2 = 66.36%, indicating that more than half of the variability of *A. annua* plant dry weight can be explained by the model. The F-test for the overall significance of the model is F (1,52) = 102.6, p< 0.001, meaning that digital biomass significantly better predicts plant dry weight than the model without predictor variables. The model error rate is 24.02% and both coefficients are significant (p<0.001). Resulting model is as follows: Dry weight = 0.3212483 + 0.0011634 · DB_d9. The model is noticeably significant because the estimated statistical power is 100% with a size effect of 1.972652.

**Figure 3:**
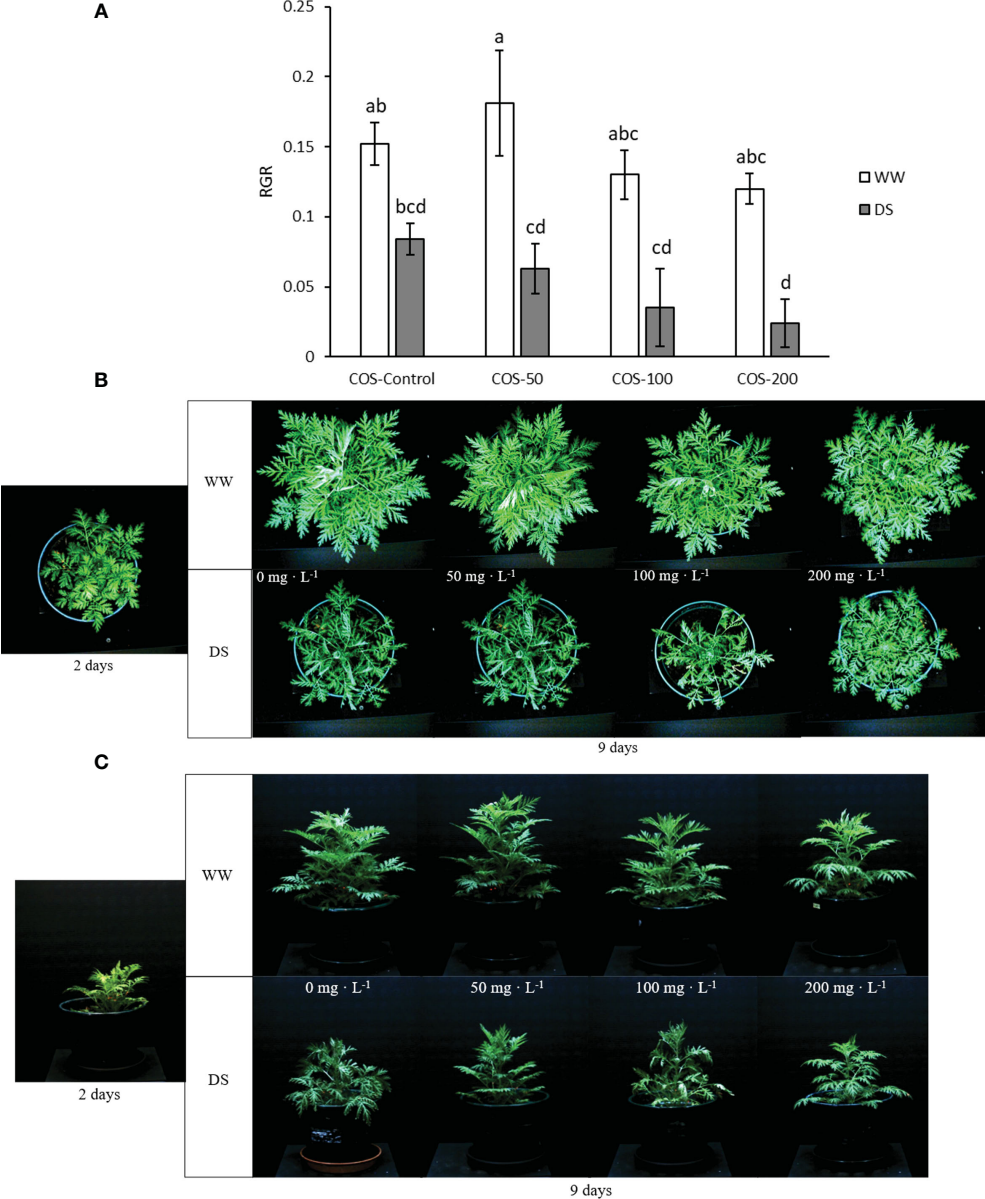
Relative growth rate **(A)** calculated with digital biomass in well-watered plants (WW) and drought-stressed plants (DS) treated with 0, 50, 100 and 200 mg·L^-1^ of COS. Different letters indicate significant differences according to the Tukey HSD test after two-way ANOVA, p< 0.05. Represented values are the mean ± SE. RGB pictures of top camara **(B)** and side camara **(C)** used to calculate the digital biomass in well-watered plants (WW) and drought-stressed plants (DS) treated with 0, 50, 100 and 200 mg·L^-1^ of COS 2 and 9 days after the start of the experiment. Representative pictures of each experimental group were selected to **(B)** and **(C)**.

Regarding growth parameters, drought stress in non-treated plants slightly reduced dry weight but it was not significantly different (**Figure 2B**). Besides, higher doses of COS slightly improved dry weight in well-watered plants but with non-significant differences with control plants. Under drought stress, COS treatment did not improve dry biomass and with 100 mg · L^-1^ dose it produced the lowest values. Thus, **Figure 3** showed a reduction of relative growth rate (RGR) calculated with digital biomass in drought-stressed plants where COS treatment made this reduction significantly different to non-treated plants under well irrigated conditions. This reduction is noticeable with higher doses. Without stress, 50 mg·L^-1^ dose slightly increased RGR and the increase in dose seems to reduce it, but the differences were non-significative. On the other side, RGR did not show significative differences in interaction factors running Two-way ANOVA (**Supplementary S.3**). Both main factors were statistically significant, but only the main factor “Irrigation condition” was significantly different with 0.144 ± 0.011 in well-watered plants and 0.054 ± 0.01 ( $\bar{\text{X}}$ ± SE).

### 3.3 Dose of 200 mg · L^-1^ of COS significantly affected fatty acids content

**Figure 4:**
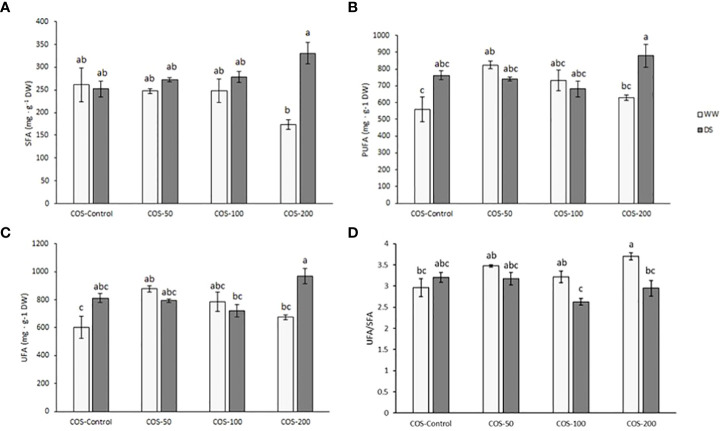
Content of saturated fatty acids **(A)**, polyunsaturated fatty acids **(B)**, unsaturated fatty acids **(C)** and UFA/SFA ratio **(D)** in well-watered plants (WW) and drought-stressed plants (DS) treated with 0, 50, 100 and 200 mg·L^-1^ of COS. Different letters indicate significant differences according to the Tukey HSD test after two-way ANOVA, p< 0.05. Represented values are the mean ± SE.

Main differences in saturated fatty acids (SFA, **Figure 4A**) were found in 200 mg · L^-1^ treatment where drought-stressed plants presented higher content than well-watered plants. Polyunsaturated fatty acids (PUFA, **Figure 4B**) and unsaturated fatty acids (UFA, **Figure 4C**) were higher in 200 mg · L^-1^ treatment under drought stress but it was only significantly different to well-watered plants with the same treatment and without treatment. No significant differences were found running two-way ANOVA in monounsaturated fatty acids (data not shown). Regarding the UFA/SFA ratio (**Figure 4D**), the results for well-watered plants treated with 200 mg · L^-1^ dose were significantly higher than for stressed-plants with this dose and control plants.

Regarding specific fatty acids, it should be highlighted the result with palmitic acid with a significative p-value in factors interaction. Thus, drought-stressed plants treated with 200 mg · L^-1^ presented higher palmitic acid levels than well-watered plants with the same treatment. Myristic acid, trans-hexadecaenoic acid and linoleic acid contents were statistically significant in the main factor ‘irrigation condition’. Thus, myristic acid was higher in stressed plants than in well-watered plants, with 61,42 ± 4,37 mg · g^-1^ DW and 48,90 ± 3,38 mg · g^-1^ DW, respectively ( $\bar{\text{X}}$ ± SE, p-value< 0.05). Similarly, linoleic acid content was significantly higher under drought stress with 213,15 ± 9,06 mg · g^-1^ DW and 181,17 ± 9,81 mg · g^-1^ DW without stress ( $\bar{\text{X}}$ ± SE, p-value< 0.05). The opposite behaviour was observed in trans-hexadecaenoic acid, where well-watered plants had higher content than drought-stressed plants with 31,46 ± 2,10 mg · g^-1^ DW and 25,62 ± 1,58 mg · g^-1^ DW, respectively ( $\bar{\text{X}}$ ± SE, p-value< 0.05). The full fatty acid profile can be found in the **Supplementary S.4**.

### 3.4 Hydrogen peroxide content increased with 200 mg·L^-1^ dose under drought stress and antioxidant enzymes machinery avoided the increase in hydrogen peroxide under well-watered conditions

**Figure 5:**
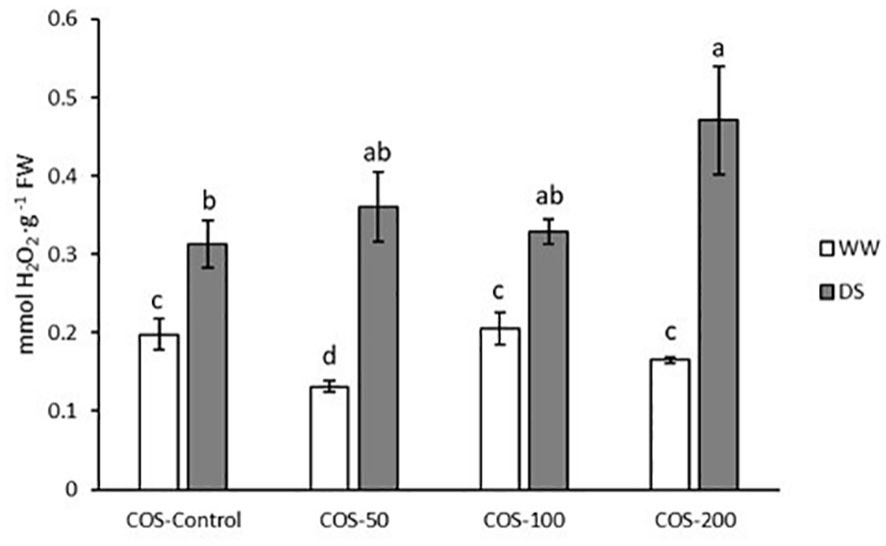
Hydrogen peroxide content in well-watered plants (WW) and drought-stressed plants (DS) treated with 0, 50, 100 and 200 mg·L^-1^ of COS. Different letters indicate significant differences according to the Kruskal-Wallis test. p< 0.05. Represented values are the mean ± SE.

Under drought stress an increased hydrogen peroxide content (**Figure 5**) was observed in contrast with well-watered plants. The highest values were recorded with 200 mg·L^-1^ dose under drought conditions which is significantly higher than for non-treated plants. In non-stressed plants, a decrease was observed with a 50 mg·L^-1^ dose.

**Figure 6:**
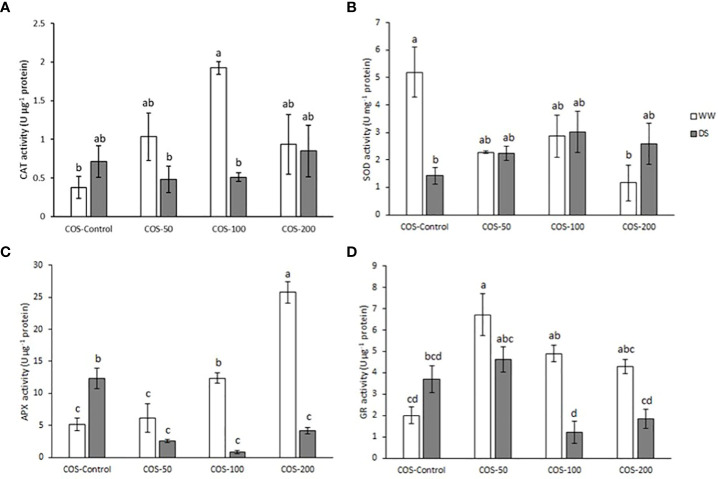
Activity of catalase **(A)**, superoxide dismutase **(B)**, ascorbate peroxidase **(C)** and glutathione reductase **(D)** in well-watered plants (WW) and drought-stressed plants (DS) treated with 0, 50, 100 and 200 mg·L^-1^ of COS. Different letters indicate significant differences according to the Tukey HSD test after two-way ANOVA, p< 0.05. Represented values are the mean ± SE.

Regarding antioxidant enzymes activity, CAT (catalase) activity (**Figure 6A**) slightly increased under drought stress, but it was not significantly different from control plants. Besides, COS treatment did not significantly modify the activity under drought stress. In well-watered plants, COS treatment increased CAT activity with significantly higher values with 100 mg·L^-1^ dose. Superoxide dismutase (SOD) activity (**Figure 6B**) significantly decreased under drought stress and COS treatment did not increase the activity. Besides, well-irrigated plants treated with COS decreased SOD activity to stressed plants level with significant differences at 200 mg·L^-1^ dose. Under drought stress, APX (ascorbate peroxidase) activity (**Figure 6C**) was significantly higher than in well-irrigated plants. Furthermore, COS treatments have the opposite result depending on irrigation conditions. On one hand, under drought stress, treatment reduced APX activity to levels of non-treated plants under well irrigation conditions. On the other hand, higher doses of COS significantly increased APX activity in well-watered plants. Similarly, drought stress increased glutathione reductase (GR) activity (**Figure 6D**) but higher doses of oligochitosan reduced it. Besides, under well-irrigated conditions COS treatment increased GR activity.

### 3.5 The application of COS treatment reduced phenols and flavonoids content under drought stress

**Figure 7:**
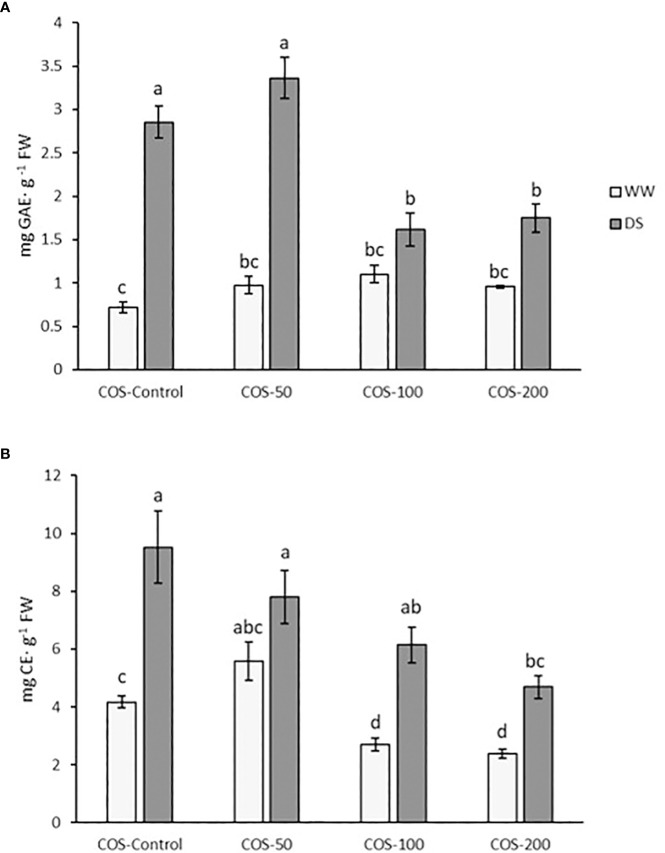
Total phenolic content **(A)** and flavonoid content **(B)** in well-watered plants (WW) and drought-stressed plants (DS) treated with 0, 50, 100 and 200 mg·L^-1^ of COS. Different letters indicate significant differences according to the Tukey HSD test after two-way ANOVA in total phenolic content and to the Kruskal-Wallis test in flavonoid content, p< 0.05. Represented values are the mean ± SE.

Regarding total phenolics content (**Figure 7A**), an increase under drought stress was observed, especially for non-treated plants and with a 50 mg·L^-1^ dose. Higher doses significantly reduced the phenol content to the level found in well-watered treated plants. Without stress, significant differences were not found between treated plants and control. Flavonoids content (**Figure 7B**) in plants treated with 50 mg · L^-1^ were slightly higher than in control plants but not significantly different. Higher doses reduced flavonoids content below control plants. Under drought stress, flavonoids were also higher, but this decreased with the COS dose increase, where the result for 200 mg · L^-1^ were similar to control plants.

### 3.6 Treatment with 200 mg·L^-1^ of COS significantly affected artemisinin production under drought stress

In the statistical analysis of artemisinin content (**Table 1**), only the main factors were significant. For the stress condition, plants under drought stress had 17.62% more artemisinin content than well-watered plants. Furthermore, drought-stressed plants treated with 200 mg·L^-1^ was significantly higher than well-watered control plants.

**Table 1:** Effect of irrigation condition, COS treatment and their interaction effect on artemisinin content.

Factors	Artemisinin content (µg · mg ^-1^ DW)
*Irrigation condition*	** (ANOVA)
Well-watered (WW)	10.06 ± 0.59 b
Drought-stressed (DS)	12.03 ± 0.54 a
*COS treatments*	** (ANOVA)
0 mg·L^-1^	10.42 ± 0.59 b
50 mg·L^-1^	11.01 ± 0.73 ab
100 mg·L^-1^	9.41 ± 0.75 b
200 mg·L^-1^	13.14 ± 0.88 a
*Irrigation condition × Chitosan treatment*	ns (ANOVA)
WW × 0 mg·L^-1^	9.77 ± 1.04 b
WW × 50 mg·L^-1^	10.97 ± 1.54 ab
WW × 100 mg·L^-1^	8.19 ± 0.99 b
WW × 200 mg·L^-1^	11.40 ± 0.93 ab
DS × 0 mg·L^-1^	10.96 ± 0.64 ab
DS × 50 mg·L^-1^	11.04 ± 0.24 ab
DS × 100 mg·L^-1^	10.87 ± 0.81 ab
DS × 200 mg·L^-1^	14.89 ± 1.16 a

Two-way ANOVA results of main factors and their interaction are indicated as: ** is p-value< 0.01 and ns is non-significative differences. Different letters indicate significant differences according to the Tukey HSD test after two-way ANOVA, p< 0.05. Represented values are the mean ± SE.

## 4 Discussion

Leaf water potential (Ψ_L_) and relative water content (RWC) are two measurements widely used to indicate plant water status. In the present study, both were significantly affected by drought stress. However, under drought stress 100 and 200 mg · L^-1^ doses improved the water potential in the plant but only 200 mg · L^-1^ increased RWC compared to non-stressed plants. Mirajkar et al. (2019) obtained similar results in sugarcane treated with normal chitosan, while COS obtained by irradiating chitosan was more effective in improving RWC.

The decrease in RWC would primarily be associated with the reduction in protoplast volume, even though a sizable portion of the water in the leaf is apoplastic (i.e., contained within the cell walls or xylem) (Sack et al., 2018). Until extremely severe tissue dryness caused embolism and drained xylem conduits, this apoplastic water would be “locked” by surface tension in the cell wall pores or xylem conduits (Sack et al., 2018). Thus, RWC represents the physiological result of cellular water deficit and evaluates the quantity of water available for leaf transpiration (El-Hendawy et al., 2019). However, Boyer et al. (2008) showed that measuring RWC with fully turgid water content when leaf floated on water can produces artefacts in RWC since this measure assumed that the osmotic potential was constant. Osmotic potential changes are caused by osmotic adjustment in cells which also modify Ψ_L_ since it consists of the sum of osmotic potential (Ψ_S_) and pressure potential (Ψ_P_). This could explain the results in 100 mg · L^-1^ which produce an osmotic adjustment that improved Ψ_L_ but produced an artefact that reduced RWC. In fact, in white clover (*Trifolium repens*) was observed that chitosan treated plants subjected to drought stress induced by polyethylene glycol (PEG) presented an increase in soluble sugar (Li et al., 2017). Soluble sugars serve as osmolytes to face osmotic stress (Afzal et al., 2021; Jiménez-Arias et al., 2021). Thus, the improvement in the hydric status of plants treated with higher doses of COS under drought stress could be due to an accumulation of soluble sugars elicited by COS.

The correlation model showed that DB and dry weight have low positive correlation but is a valid model to estimate dry weight from DB. However, DB is calculated from the area of fresh plants which also showed changes in water content. It is important to take into account that foliar water content is intimately related to leaf shape, angle and structure (Chaves et al., 2002). These affect the area calculated from the phenotyping platform. Huang et al. (2019) showed that leaf fresh weight had a stronger relationship with the area than leaf dry weight and highlighted the importance of foliar water content in leaf growth and form. This should be considered for future research.

Water scarcity has been widely reported to reduce growth (Bistgani et al., 2017; Pirbalouti et al., 2017; Alizadeh et al., 2020; Ahluwalia et al., 2021). Specifically, *A. annua* presented a growth reduction with the increase of drought stress (Yadav et al., 2014; Soni and Abdin, 2017). The present study showed a reduction in dry weight and RGR under drought stress where COS treatment did not significantly improve growth parameters. Without stress, chitosan (Ait Barka et al., 2004; Mondal et al., 2016; Harfoush et al., 2017) and degraded chitosan (such as COS) have been reported to improve growth in different species (Dzung et al., 2011; Rahman et al., 2013; Dzung et al., 2017; Muley et al., 2019). However, in *A. annua* plants under well-irrigated conditions, differences between treatments were not statistically significant. This is in accordance with results reported by Lei et al. (2011), where the foliar application of 100 mg · L^-1^ of chitosan did not produce significant differences in dry weight and height over 16 days. Besides, Bittelli et al. (2001) found that chitosan foliar treatment reduced biomass and yield in pepper plants (*Capsicum* sp.) but the differences were not significantly different. Similar results were found in summer savory (*Satureja hortensis* L.), where the differences in dry weight were not significantly different (Alizadeh et al., 2020). It seems to indicate that in these species, triggered strategies to face drought stress were not enough to improve the biomass. Thus, in the present results, COS treatment at a higher dose improved Ψ_L_ and RWC under drought stress but did not significantly improve dry weight and RGR.

In plants, fatty acids are broadly dispersed on the cell surface and serve to prevent moisture and heat loss, and they are linked to cell recognition specificity and tissue immunity (Gu et al., 2020). Thus, alterations in the fatty acid profile could potentially serve as a membrane adaptation mechanism to cope with prolonged stress (Qureshi et al., 2013).

The SFA results show an interesting, oppositive response in well-watered plants and under drought stress, particularly at 200 mg · L^-1^ COS dose. Thus, while the SFA decreased for WW plants, it increased for DS plants. This is due to changes in the levels of myristic acid (C14:0) and palmitic acid (C16:0). In particular, higher COS doses increased palmitic acid content under drought stress, probably due to inhibition of lipid desaturase activities as has been reported in Kentucky bluegrass (*Poa pratensis* L.) (Xu et al., 2011). Concerning the increase of myristic acid levels under drought stress, Qureshi et al. (2013) also found that its levels increased under other osmotic stress (salt stress). Authors suggested that protein myristoylation plays a role in the response to this stress since it is a crucial process in membrane targeting and signal transduction. In fact, Sainas et al. (2018) describe this reaction as a lipid modification that involves the addition of myristic acid to the N-terminal glycine of a subset of proteins and promote their binding to cell membranes for a range of biological functions.

On the other hand, the increase of UFAs, including PUFA, in drought-stressed plants treated with 200 mg · L^-1^ COS dose is mainly influenced by linoleic acid (C18:2) and slightly by α-linolenic acid (C18:3). The present study showed a higher linoleic acid (C18:2) content in drought stressed plants. The increase of linoleic acid under salt stress in *A. annua* (Qureshi et al., 2013) and drought stress in Kentucky bluegrass (Xu et al., 2011) was related to changes of desaturases activity. Furthermore, Tang et al. (2012) found that higher levels of C18:2 and C18:3 were related to lower susceptibility to cold stress. Besides, under drought stress, it has been reported an increase in UFA in coconut (*Cocos nucifera* L.) (Repellin et al., 1997), grapevine (*Vitis vinifera*) (Toumi et al., 2008), cork oak (Sebastiana et al., 2018; Sebastiana et al., 2019) and in *Arabidopsis thaliana* (Gigon et al., 2004).

In well-watered plants treated with 200 mg · L^-1^ dose, the UFA/SFA ratio is significantly higher than in control plants. This indicated lower SFA with respect to UFA content. Fatty acids are the primary components of cellular membranes, and changes in unsaturation levels and FA composition are directly related to plant resistance to various stresses, including drought (Zhang et al., 2005). Due to the non-linear nature of fatty chains containing double bonds, highly unsaturated fatty acids are less densely packed into a membrane, resulting in a less rigid membrane structure. (Lehninger, 1977; Cyril et al., 2002). Thus, the 200 mg · L^-1^ COS dose made the membrane less rigid under well-irrigation condition. This suggests that maintaining higher fatty acid unsaturation levels could favour proper membrane fluidity, required for several membrane-dependent processes for plant stress adaptation (Xu et al., 2011; He and Ding, 2020). It has been reported that under conditions of drought stress, chitosan can stabilise membranes through an increase of antioxidant activity, as shown for apple tress (*Malus sieversii*), potato (*Solanum tuberosum*) and *Thymus daenensis* (Yang et al., 2009; Jiao et al., 2012; Bistgani et al., 2017). Antioxidant activity prevents the lipid peroxidation that causes membrane damage. In present study, the increase in the activity of the antioxidant enzymes activity in well-watered plants treated with COS may promote membrane stability. However, this improvement was not observed under drought stress. This likely results from damage caused by ROS, which react with the double bonds of fatty acids, since unbalanced ROS can easily oxidize PUFA and trigger lipid peroxidation (Vistoli et al., 2013; Alché, 2019). Nevertheless, the reduction of membrane fluidity limits free radicals diffusion and thus reduces lipid peroxidation in membranes (Mukhtar Ahmed et al., 2020).

There are not many works about the *A. annua* fatty acid profile and its changes under stress. Carvalho et al. (2011) determined the fatty acids profile in different species of *Artemisia* spp. and as in the present work, the major fatty acids identified were α-linolenic acid followed by linoleic acid and palmitic acid. Qureshi et al. (2013) obtained similar results at 100 and 130 days after sowing (DAS). At 160 DAS, authors found higher content of linoleic acid followed by α-linolenic acid and palmitic acid. These differences are likely due to different growth conditions and physiological stages.

Chitosan treatment induced the production and accumulation of H_2_O_2_ in several species (Lin et al., 2005; Li et al., 2009; Mejía-Teniente et al., 2013). However, in our results COS treatment did not increase H_2_O_2_ in well-watered plants. In fact, the 50 mg · L^-1^ dose significantly decreased it while higher doses maintained similar levels than non-treated plants under well-watered conditions. These results could be due to the increase in CAT, APX and GR activity, since these enzymes play an important role in H_2_O_2_ scavenging.

Some authors have shown that hydrogen peroxide elicited by chitosan treatment has a key role in stomata closure signalling and detected accumulation of H_2_O_2_ in guard cells (Iriti et al., 2009; Li et al., 2009; Srivastava et al., 2009). Thus, the authors showed that chitosan has antitranspirant activity due to the H_2_O_2_ accumulation and it is this feature the main reason to propose it to face drought stress. However, Iriti et al. (2009) and Mirajkar et al. (2019) point out that these results are valid under optimum conditions, but under drought stress the response can be different. In fact, Mirajkar et al. (2019) reported that COS treatment decreased H_2_O_2_ levels and increased SOD activity under drought stress. In contrast, the present study found an increase in H_2_O_2_ content under drought stress with higher doses of COS. Also, in COS-treated plants under drought stress, CAT and SOD activity did not increase while APX and GR decreased. This decay in the antioxidant enzyme machinery could be caused by high stress levels that damaged it. Thus, it must be pointed out that the present study was carried out after 9 days of stress while the mentioned anti-transpirant experiments were carried out in the first stages of stress. Nevertheless, it could be possible that under severe drought conditions the observed increase in H_2_O_2_ induced abscisic acid (ABA) signalling. This plant hormone plays a main role in stomata closure and Zhang et al. (2001) showed that H_2_O_2_ is involved in ABA signalling and how exogenous catalase partially prevents stomatal closure. Since this is a mechanism to avoid water loss, H_2_O_2_ production could be one of the mechanisms involved in the improvement of RWC and Ψ_L_ with 200 mg · L^-1^ dose.

Regarding the content of phenolic compounds, these natural products are abundantly found in the Plant Kingdom and play an active role in protection processes against biotic and abiotic stresses (Naikoo et al., 2019). As an antioxidant defence, the content of these secondary metabolites usually increases under drought stress, as was reported for different species (Quan et al., 2016; Mohamed and Latif, 2017; Sarker and Oba, 2018; Gharibi et al., 2019; Kirova et al., 2021). Those results are in agreement with these obtained in the present work, where total phenolics and flavonoids increased under drought stress.

There are many reports on the increase of phenols and flavonoids under chitosan elicitation (Khan et al., 2019; Hawrylak-Nowak et al., 2021; Kahromi and Khara, 2021; Bavi et al., 2022). Besides, in sage (*Salvia officinalis* L.) extracts growing under reduced irrigation conditions and sprayed with chitosan, the total content of phenolic and flavonoids increased (Vosoughi et al., 2018). However, in the well-watered plants of the present study, the increase in phenols was not significant compared to the control. In fact, higher doses decreased flavonoids. Furthermore, their content decreased in drought-stressed plants under COS treatment, particularly at higher doses. A possible explanation is found in some reports about the toxic effects of high chitosan doses on cell cultures (Cabrera et al., 2006; Wang et al., 2008; Singh, 2016). Thus, chitosan treatment elicited ROS production, which in turn can promote the synthesis of defence compounds such as phenols, flavonoids, enzymes, etc. (Gechev et al., 2002). However, ROS can also cause cell damage and cell death when the production is excessive (Lawton and Lamb, 1987). Cabrera et al. (2006) and Singh (2016) related a decrease in the level of metabolites at higher chitosan and COS doses due to an imbalance of oxidative stress molecules production. This excess of ROS, caused by a high elicitor signal, disturbs the synthesis of defensive compounds, and the cell can no longer repair the damage. This is probably happening in the present study when drought-stressed plant underwent COS treatment, namely, cell damage affected phenolic and flavonoid synthesis. On the other hand, in well-irrigated plants, oligochitosan treatment increased CAT, APX and GR activity allowing low hydrogen peroxide content. This suggests a COS activation of antioxidant enzymes, but not the non-enzymatic antioxidant defence.

Drought stress was the main factor that affected the content of metabolites, and increased artemisinin production. Soni and Abdin (2017) reported that during the plant vegetative stage, artemisinin content was lower for drought-stressed plants control. Yadav et al. (2014) also reported a decrease in the artemisinin content under drought stress. However, in these previous works, the stress level was constant over time while in the current work there was a progressive decrease in SWC (soil water content). In another report, Vashisth et al. (2018) found a decrease in the artemisinin content under drought stress but this was not statistically significant. Marchese et al. (2010) studied artemisinin content withholding watering until 86 h and only found a significant increase at 38 h. As shown in **Table 1**, well-watered and stressed non-treated plants gave similar results, except for the 200 mg · L^-1^ dose, which increased artemisinin under drought stress.

Foliar application of 100 mg · L^-1^ of chitosan increased artemisinin content 48 h after treatment (Lei et al., 2011). Since chitosan increased H_2_O_2_ and O_2_^-^ content, authors related this result to a decrease in the artemisinin precursor, dihydroartemisinic acid. The hypothesis is that this precursor acted as ROS quencher, yielding artemisinin as end-product (Wallaart et al., 2000; Lei et al., 2011). Although dihydroartemisinic acid oxidation has been mainly related to O_2_^−^, it has also been shown that H_2_O_2_ plays a major role in artemisinin synthesis (Kam and Yap, 2020). In this way, (Mannan et al., 2010) reported that dimethyl sulfoxide (DMSO) produced an increase in H_2_O_2_ and artemisinin levels. To verify that both events are related, authors added to *in vitro* cultures L-ascorbic acid, a natural scavenger of ROS, and observed that both H_2_O_2_ and artemisinin production were greatly reduced. Thus, an increase in ROS likely resulted in higher artemisinin content in DS plants treated with 200 mg · L^-1^ COS.

Present study obtained non-significant differences in well-watered plants. This is in agreement with the studies by Yin et al. (2012b), where the increase in artemisinin content was similar in control and COS-treated plants. Therefore, the authors’ conclusion is that the application of elicitors may not be universally effective in increasing the yield of artemisinin from *A. annua* plants. Besides, the present study showed that CAT, APX and GR activity increased with oligochitosan treatment in well-irrigated plants allowing low ROS content. Thus, due to the crucial role of ROS in artemisinin production as mentioned before, it appears that well-watered plants utilise antioxidant enzymes instead of dihydroartemisinic acid to quench ROS, and artemisinin synthesis is not elicited.

However, chitosan and their derivatives characteristics such as degree of acetylation and polymerization, functional group availability or molecular weight can change their biological response (Harish Prashanth and Tharanathan, 2007; Mirajkar et al., 2019; Mukhtar Ahmed et al., 2020). This, as well as the studied specie, can affect the treatment response. Thus, further studies on the characteristics of chitosan and its biological effect are required since it showed to be not a universal elicitor. Ahmad et al. (2020) reviewed signalling in the production of plant secondary metabolites elicited by oligosaccharides and highlight the progress in this area. Thus, studies such as Zou et al. (2015); Zou et al. (2017); Mirajkar et al. (2019) and Muley et al. (2019) could allow a deep understanding of the mode of action and the relation between the structures and characteristics of chitosan and their derivative with their activity.

## 5 Conclusion

*Artemisia annua* has anti-malarial, anti-tuberculosis, anti-cancer and anti-microbial compounds with great commercial and medicinal relevance (Soni et al., 2022). *A. annua* plants are the best source of artemisinin production, but still low yield are obtained (0.5-0.8%), and thus an increase in production is a critical issue (Soni et al., 2022).

The effect of COS on artemisinin production in well-watered and drought-stressed plants was addressed in the present work, as well as *A. annua* growth, metabolism and water management. Thus, under drought stress, COS did not counteract the reduction in growth at any concentration, but higher doses improved plant water status, probably due to osmotic regulation and stomata closure mediated by ABA and H_2_O_2_ signalling. Furthermore, combined COS treatment and drought stress induced damage to antioxidant enzyme defence (especially APX and GR) and reduced the content of phenols and flavonoids. This boosted ROS production and led to an increase in artemisinin content in DS plants at a 200 mg·L-1 COS dose, probably due to dihydroartemisinic acid oxidation. In contrast, under well-watered conditions, COS did not enhance the growth of *A. annua*, and since the antioxidant enzyme machinery was not damaged, it prevented artemisinin elicitation.

These results highlight the significant role of ROS in artemisinin synthesis and suggest that COS application has the potential to increase artemisinin yield in crop production, even under drought conditions. However, a deeper understanding of COS characteristics and the elicitation process of artemisinin is necessary to achieve cost-effective production.

## Data availability statement

The raw data supporting the conclusions of this article will be made available by the authors, without undue reservation.

## Author contributions

DJ-A, JMdS, AB and AG-G designed the idea of the manuscript. AG-G performed the experiments with participation of AM, EF, and RCdC. All authors contributed to the article and approved the submitted version.
