# Supplementary material for: The use of chitosan oligosaccharide to improve artemisinin yield in well-watered and drought-stressed plants
Source: Front Plant Sci. 2023 Jun 2;14:1200898. doi: 10.3389/fpls.2023.1200898 (PMC10272596; doi:10.3389/fpls.2023.1200898)
Supplement: Supplementary file 1 [file DataSheet_1.docx]

Supplementary Material

Article Title

First Author*, Co-Author, Co-Author

*** Correspondence:** Corresponding Author: email@uni.edu

# Supplementary Figures and Tables


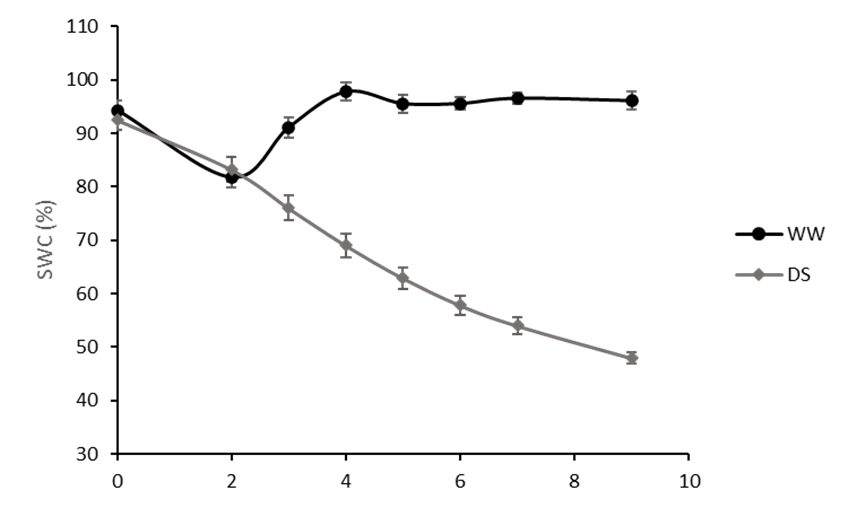


**Supplementary Figure (S.1).** Soil water content (SWC) in well-watered (WW) and drought-stressed (DS) plants over 9 days. Represented values are the mean ± SE.

**
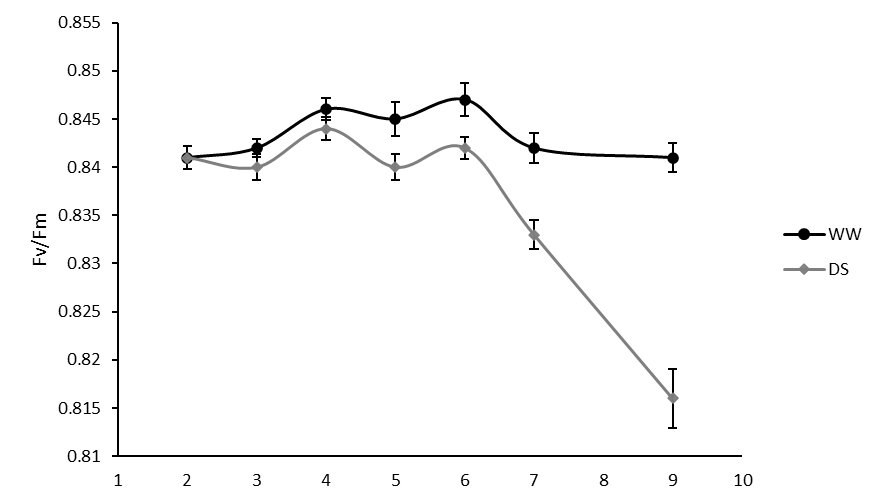
**

**Supplementary Figure (S.2).** The maximum photosystem II quantum efficiency (Fv/Fm) in well-watered (WW) and drought-stressed (DS) plants over 9 days. Represented values are the mean ± SE.

**Supplementary Table (S.3).** Effect of irrigation condition, COS treatment and their interaction effect on RGR calculated with digital biomass. Two-way ANOVA results of main factors and their interaction are indicated as: * is p-value < 0.05, *** is p-value < 0.001 and ns is non-significative differences. Different letters indicate significant differences according to Tukey HSD test after two-way ANOVA, p < 0.05.

| **Factors** | **RGR** |
| --- | --- |
| *Irrigation condition* | *** (ANOVA) |
| Well-watered (WW) | 0.144 ± 0.011 a |
| Drought-stressed (DS) | 0.054 ± 0.010 b |
| *Chitosan treatments* | * (ANOVA) |
| 0 mg·L^-1^ | 0.118 ± 0.014 a |
| 50 mg·L^-1^ | 0.112 ± 0.025 a |
| 100 mg·L^-1^ | 0.083 ± 0.022 a |
| 200 mg·L^-1^ | 0.08 ± 0.017 a |
| *Irrigation condition × Chitosan treatment* | ns (ANOVA) |
| WW × 0 mg·L^-1^ | 0.152 ± 0.015 ab |
| WW × 50 mg·L^-1^ | 0.181 ± 0.038 a |
| WW × 100 mg·L^-1^ | 0.130 ± 0.035 abc |
| WW × 200 mg·L^-1^ | 0.120 ± 0.011 abc |
| DS × 0 mg·L^-1^ | 0.084 ± 0.011 bcd |
| DS × 50 mg·L^-1^ | 0.063 ± 0.018 cd |
| DS × 100 mg·L^-1^ | 0.035 ± 0.028 cd |
| DS × 200 mg·L^-1^ | 0.024 ± 0.017 d |

**Supplementary Table (S.4).** Fatty acid profile from A. annua leaves (mg · g^-1^ DW) in well-watered (WW) and drought-stressed plants (DS) treated with 0, 50, 100 and 200 mg·L^-1^ of COS. Different letters indicate significant differences according to the Tukey HSD test after two-way ANOVA, p < 0.05. Represented values are the mean ± SE.

| Fatty acid | | WW | | | | DS | | | |
| --- | --- | --- | --- | --- | --- | --- | --- | --- | --- |
|  |  | C0 | C50 | C100 | C200 | C0 | C50 | C100 | C200 |
| Myristic acid | C14: 0 | 52.11 ± 8.43 | 56.05 ± 3.88 | 41.21 ± 8.00 | 43.62 ± 5.45 | 59.16 ± 6.70 | 54.74 ± 2.29 | 61.15 ± 5.93 | 69.02 ± 15.61 |
| Palmitic acid | C16:0 | 167.27 ± 20.25 (ab) | 163.26 ± 4.39 (ab) | 169.50 ± 15.26 (ab) | 116.09 ± 4.04 (b) | 170.97 ± 11.92 (ab) | 186.37 ± 2.57 (ab) | 192.56 ± 11.33 (a) | 216.25 ±13.94 (a) |
| Trans-hexadecanoic acid | C16:1t | 31.46 ± 5.35 | 32.20 ± 1.35 | 34.54 ± 5.39 | 25.26 ± 0.42 | 27.89 ± 3.49 | 24.71 ± 1.79 | 20.871 ± 3.12 | 30.131 ± 2.91 |
| Stearic acid | C18:0 | 39.67 ± 7.81 | 27.69 ± 5.12 | 41.73 ± 6.51 | 29.53 ± 3.42 | 29.22 ± 5.05 | 29.54 ± 2.78 | 26.73 ± 1.32 | 27.24 ± 2.21 |
| Oleic acid | C18:1 | 22.67 ± 0.68 | 20.38 ± 3.54 | 24.92 ± 4.85 | 16.87 ± 1.31 | 28.03 ± 5.85 | 28.68 ± 1.99 | 20.01 ± 2.51 | 22.41 ± 4.95 |
| Linoleic acid | C18:2 | 187.67 ± 30.74 | 199.51 ± 2.52 | 192.41 ± 16.38 | 145.10 ± 10.23 | 201.09 ± 5.91 | 193.60 ± 4.67 | 218.89 ± 21.51 | 234.57 ± 22.24 |
| α-Linolenic acid | C18:3 | 507.30 ± 85.11 | 604.03 ± 31.13 | 511.89 ± 46.80 | 446.00 ± 8.31 | 535.32 ± 17.34 | 515.96 ± 12.56 | 458.49 ± 31.64 | 607.00 ± 49.18 |
| Others |  | 24.43 ± 6.98 | 27.29 ± 8.75 | 31.60 ± 4.33 | 30.56 ± 3.14 | 35.90 ± 6.91 | 35.03 ± 6.10 | 23.36 ± 3.20 | 42.32 ± 9.50 |
